# The functional barometer –a self-report questionnaire in accordance with the international classification of functioning, disability and health for pain related problems; validity and patient-observer comparisons

**DOI:** 10.1186/1472-6963-14-187

**Published:** 2014-04-24

**Authors:** Jan-Rickard Norrefalk, Elisabeth Svensson

**Affiliations:** 1 Division of Rehabilitation Medicine, Department of Public Health Sciences, Karolinska Institutet, Stockholm, Sweden; 2 Department of Statistics, Örebro University, Örebro, Sweden

**Keywords:** Disagreement measures, ICF, Ordinal data, Pain, Patient-professional disagreement, Questionnaire, Rehabilitation, Validity

## Abstract

**Background:**

The International Classification of Functioning, Disability and Health, (ICF) provides a unified, international standardized framework for describing and classifying health and health-related functioning and disability. Based on an ICF core sets the Functional Barometer, (FB), was developed for assessments of perceived pain-related problems with functions, activity and quality-of-life by patients suffering from long-term pain. The aim was to evaluate the construct validity, and to compare the assessments of a patient’s problems from the perspectives of the patient and of the examining professional when using the validated FB and corresponding ICF-classification form, respectively.

**Methods:**

Patients with long-term pain for more than 3 months that visited one of four pain clinics during specified time periods of data collections were eligible. The self-report Balanced Inventory for Spinal disorders was used for validation of the FB. Correspondingly to the validated FB an ICF-classification form for professional’s assessment was developed. The data sets for these inter-scale and the patient-professional comparisons were collected adjacent to the clinical examination. By the statistical method used for evaluation of the pairs of ordinal data presence of systematic disagreement was identified and measured separately from additional individual variations.

**Results:**

The validation process resulted in a revised FB(2.0) that meets the requirements of good construct and content validity. The professionals’ ICF-classifications of the patients’ problems disagreed with the patients’ assessments on the FB(2.0); the percentage agreements ranged from 18% to 51%. The main reason was that the practitioners systematically underestimated the patients’ levels of problems but the different professionals contributed also to a large individual variability (noise) in assessment.

**Conclusions:**

This study presents an ICF-based validated self-report questionnaire, The FB, to be used for identifying and describing pain-related problems with current functioning, activities and quality-of-life as perceived by patients suffering from long-term pain. The strong evidences of underestimation of the patients’ problems and the variability in the professionals’ ICF-classifications demonstrated the importance of describing the patient’s problems both from the patient’s and the professional’s perspective beneficial for the patient’s rehabilitation.

## Background

Pain is a subjective perception that is linked to various co-morbidities and mental disorders [1-3]. Musculoskeletal pain is one of the most common reasons for a visit to primary health care in Scandinavia, where 20-50% of those seeking health care experience long-term pain [4,5]. Long-term pain is also a major reason for prolonged sick leave and early retirement, thereby causing high costs for the national insurance system [6]. Furthermore perceived long-term pain will affect an individual’s life, spare time, economy, psychosocial well-being and capacity for work [7].

The multi-dimensional characteristics of perceived pain is evident by the diversity of operational definitions of pain and pain-related disability ranging from assessments on one or more rating scales with a discrete number of ordered alternatives or on visual analogue scales to electrical pain threshold assessments [8]. Visual Analogue Scales [9], the Modified Somatic Perception Questionnaire [10], the Disability Rating Index [11], the Multidimensional Pain Inventory [12-14], and the Hospital Anxiety and Depression scale [15] are all included in the Swedish quality registry for pain rehabilitation to facilitating comparisons of pain rehabilitation programs in Sweden [16].

International comparisons would be facilitated by questionnaires referring to the World Health Organization’s International Classification of Functioning, Disability and Health, (ICF) [17]. The ICF was developed to provide a unified, international and standardized framework to describe and measure health and disability. Functioning and disability refer to three key component; body function and structure (impairment), activities (limitation), and participation (restriction) and these components may interact with health conditions and personal and environmental factors [17-19]. According to the conceptual context the ICF have 1424 well-defined categories in a four-level hierarchical structure representing constructs, and items that are the indicators for estimating the variation in the construct [19]. The first level categories are called chapters.

On the other hand in the context of the measurement process for development of questionnaires that we use in this paper, the ICF has a hierarchical structure of well-defined variables and sub-variables. The first level category, an ICF chapter, is often called dimension, especially in multi-dimensional questionnaires. Each of the variables can be operationally defined to being measured by an item, for example a question suitable for the specific implementation of the ICF [20,21]. Assessment of each single item in a self-report questionnaire is made on a five-point scale, a so-called ICF qualifier, and the categories being: no, mild, moderate, severe, complete problem. Having a problem may mean impairment, limitation, restriction or a barrier depending on which of the three key components of functioning and disability being estimated [18,19,22].

Content comparison studies have shown that health-related quality-of-life instruments can be mapped to the ICF, and Chieza and Stucki [17] consider also that quality-of-life can be regarded as an individual’s perception of health and health-related aspects of well-being.

The ICF categories/variables can be used as building blocks for creating ICF based core sets addressing different implementations. These core sets can be the starting point for development of clinical or self-report questionnaires for estimating various aspects of functioning in specified studies. ICF core sets have for example been developed in acute medicine [23], in physical and rehabilitation medicine [24] or more specified in patients with chronic conditions [25], fibromyalgia [26], chronic widespread pain [27], or spinal cord injury [28].

Many ICF variables are suitable for expert’s assessments and of patient-oriented instruments for clinical practice. Rauch et al. [22] describe how the ICF tools can be used in the entire rehabilitation process both by the patient and the professionals involved in the care of the patient. Assessment, assignment, intervention and evaluation were the proposed steps of this rehabilitation process. The purpose of the assessment is to describe the patient’s limitation in functioning and identify the needs both from the patient’s and the health professional’s perspective.

With the Swedish version of the WHO Guidelines and the ICF core sets for chronic widespread pain as base a self-report questionnaire for patients suffering from long-term pain conditions, the Functional Barometer (FB) was developed. Correspondingly an ICF-classification form for assessment of the patient’s problems from the professional’s perspective based on clinical examination was composed [18,27,29]. The focus of this study is to validate the operational definitions of the items of the FB. Furthermore, the professional’s understanding of a patient’s pain-related functioning state is important for the rehabilitation process for decisions about the patient’s needs for support in daily life to facilitate the patient’s rehabilitation [27,29].

To our knowledge this is the first time ICF core sets have been used for comparing the assessments of a patient’s functioning state from the patient’s and a professional’s perspectives. The use of standardized ICF assessments by the same problem scales (ICF Qualifiers) provide additional aspects of the patient’s functioning and an opportunity to describe possible differences in estimation and perception between patients and professionals. The statistical approach used in this study makes it possible to evaluate such differences in assessments and explain the observed disagreement in terms of systematic and individual disagreements, respectively. Presence of systematic disagreement refers to the group of professionals and patients which is important information that could have an impact on the rehabilitation plan for the patients [20,30-32].

Quality control of questionnaires, item scales and assessments involves the three components: validity, reliability and responsiveness, each having sub-concepts referring to specific contexts, types of studies and applications. Quality control is an ongoing process [20,33]. Evaluations of inter- and intra- individual reliability are study specific and must be considered when choosing a questionnaire to a study. The responsiveness to change refers to studies of treatment effects or other follow-up studies. Validity refers to the ability of a questionnaire to measure what it is intended to measure and involves different aspects on the quality of the operational definitions of those ICF variables that are included in the FB. Content and construct validity refer to the choice of variables, the operational definitions and will be generally valid. Content validity refers to the completeness of the questionnaire in the coverage of important areas [20,34-36]. The content validity of the FB variables is agreed to the unified and standardized framework of ICF variables and to the ICF core sets used [17]. Construct validity refers to the consistency in assessments between items that intend to measure the same or similar theoretical variable, and is an umbrella term for terms like convergent, descriptive, factorial, translation validity and parallel reliability [20].

In this study, the assessments on the original version of the Functional Barometer (1.0) were compared with assessments on corresponding items of the Balanced Inventory for Spinal Disorders (BIS) [35], which is a validated self-report questionnaire that measures to what extent pain affect the physical and mental health, social life and quality-of-life.

The aim of this paper is firstly to evaluate the construct validity and to present a validated Functional Barometer. Secondly, to investigate the agreement between the patients’ self-rated problems on the validated FB and the professionals’ ICF-classification of corresponding problems of the patient.

## Methods

### The questionnaires

The Functional Barometer, FB(1.0) was developed by the author (JRN) to meet the need for a self-report questionnaire for assessments of perceived pain-related problems by patients suffering from long-term pain condition that was experienced in discussion with patients and professionals in pain management, general medicine and occupational health. Relevant ICF categories/variables of the components body function and structure, and activities and participation were identified from the ICF core sets for chronic widespread pain in accordance with the Swedish version of the ICF Checklist [18,27,29]. The variables were approved by multimodal pain rehabilitation professionals in consensus as being suitable for providing valuable information about the patient’s pain-related problems in daily life for description of patient’s problems and resources, monitoring, rehabilitation and documentation of the rehabilitation process. The FB has 12 specified variables covering body function and activities/participation and one additional optional item variable for the patient to assess, 12 quality-of-life variables, and four items of pain (Table 1). All items are assessed by a verbal descriptive problem scale, the same as the so called ICF qualifier, the five categories being *no, slight, moderate, major,* and *total problems.*

**Table 1:** International Classification of Functioning, Disability and Health (ICF) variables included in the Functional Barometer (FB)

**Do you have problem because of your pain with …….. Scale categories: no, slight, moderate, major, total problem**		
**FB no**	**ICF-code**	**Function/Activity**
1	d 540	Dressing
2	b 710	Joint mobility
3	b 730	Muscle strength
4	b 740	Endurance
5	d 450	Walking
6	d 4551	Walking in stairs
7	d 4153	Keeping posture
8	d 649	Making the bed
9	d 640	Ordinary housework
10	d 430	Lifting/carrying things
11	d 4751	Driving a car
12	d 470	Using transportation
**Do you have problem because of your pain with….. Scale categories: no, slight, moderate, major, total problem**		
**FB no**	**ICF code**	**Quality-of-life**
14	b 134	Sleeping
15	b 130	Energy
16	b 160	Concentration
17	d 240	Stress. psychological demands
18	b 152	Emotional functions
19	b 535	Gastro-intestinal functions
20	d 920	Leisure time
21	d 760	Family relation
22	d 770	Partner relationship
23	d 750	Contact with friends
24	d 750	Self-support
25	d 850	Managing pay-work
		**Perceived pain**
26	b 280	Pain just now
27	b 280	Pain the last week
28	b 280	The mildest pain
29	b 280	The worst pain

The item number of the FB and corresponding ICF-code is shown.

Correspondingly an ICF-classification form for assessment of the patient’s problems from the professional’s perspective was constructed.

The BIS is a validated multi-dimensional questionnaire with 18 items for assessments of perceived level of pain, the level of limitations in pain-related body functions/activities and in quality-of-life [35]. All item-scales have five ordered categories with the verbal descriptive categories formulated to fit the different items. The verbal ordered categories of the limitation scale are *not at all, negligible, moderate, quite a bit*, and *very much.* This means that the two questionnaires have somewhat different operational definitions of the variables of interest, but could be used in similar applications.

### Study designs and patients

Since the FB is addressed to patients with long-term pain when visiting different clinical settings and professionals a variety of patients and professionals were included. Our study design by consecutively inviting all patients with long-term pain for more than 3 months that visit a pain clinic in central Sweden during a specified time period reflects the reality in which the FB will be used. Statistical simulation studies and experiences gained from clinical studies of inter-scale and inter-rater comparisons have shown that a set of 20 paired data will provide enough power for identifying and measure evidence of systematic disagreement and individual variations between assessments [20,37]. However, to ensure heterogeneity in the participating patients the goal was to include 80 and 40 eligible patients during the planned data collection time frames of the two studies, respectively.

Data for the evaluation of construct validity of the FB(1.0) regarding the operational definitions of the items and the order consistency between the assessments on the FB(1.0) and the BIS were collected between autumn 2010 and spring 2011. After informed consent, 89 patients who visited one the four pain clinics involved in this part of the study filled out the FB(1.0) and the BIS questionnaires in the waiting room before the medical examination by a professional.

Data for the patient-observer comparisons between the assessments on the revised validated FB(2.0) were collected between December 2011 and February 2012. The 41 patients who visited the pain clinic involved during this period and consented to participate after information filled out the FB(2.0) before the medical examination. Eleven multi modal team professionals, well experienced on the ICF-classification procedure [18,29] were informed and involved as observers at the clinic. Each patient was examined at the pain clinic by the observer, who filled out the ICF-classification form independently of the patient’s assessments on the FB(2.0).

The study was conducted in accordance with the Helsinki Declaration and approved by the Regional Ethical Review Board in Uppsala (2010/350 and 2011/381).

### Statistical methods

**Figure 1:**
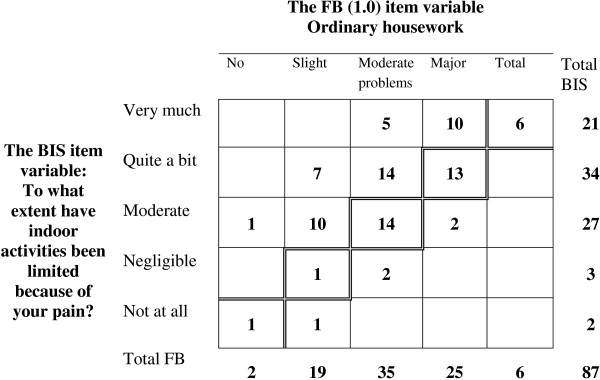
**The frequency distribution of the pairs of assessments on the Functional Barometer, FB(1.0) of perceived problems with ordinary housework, and on the Balanced Inventory for Spinal disorders (BIS) of perceived pain-related limitations in indoor activities.** The agreement diagonal is marked.

Assessments on rating scales generate ordinal data, which means that the values represent only an ordered categorical level and not a numerical value in a mathematical sense, even when the assessments are numerically coded. Therefore statistical methods applicable to data from scale assessments differ from the traditional methods for quantitative data, especially when dealing with paired data [38-43]. The median approach, Md (Q_1_;Q_3_) and bar charts were used to describe item response profiles of the group, when applicable. The frequency distribution of pairs is shown by a square contingency table, the agreement diagonal being oriented from lower left to upper right corner [20,30,31,35]. The percentage agreement, PA, was calculated. The two sets of marginal frequencies describe the distribution of each set of assessments, see Figure 1.

Both the inter-scale and the patient-observer comparisons were investigated by a non-parametric statistical method for paired ordinal data that makes it possible to identify and measure systematic disagreement separately from disagreement caused by individual variations. These two sources of an observed disagreement provide different information of the quality of data, since systematic disagreement refers to the group, and individual variations are related to individual pairs of assessments.

The evaluation of the construct validity refers to the level of order-consistency between the FB(1.0) items and the corresponding or similar items in the BIS. This is a much stronger requirement of validity than is the measure of association between the assessments expressed as the Spearman rank-order correlation coefficient, which is also calculated [29]. A high level of order-consistency means that most patients will keep their ordering relative each other in the two of assessments but not necessarily agree in categories [44]. Then the two sets of marginal distributions differ, which indicate presence of systematic disagreement. The measure of Relative Position, RP, expresses the extent to which the marginal distribution of assessments on the BIS is systematically shifted towards higher categories than the marginal distribution of assessments on the FB(1.0) rather than the opposite. A theoretical description of this systematic shift in position is the difference between the probabilities Prob(FB < BIS)-Prob(BIS < FB). Possible values of RP range from -1 to 1, and RP is positive when higher scale categories are more frequently used in the assessments on the BIS than on the FB(1.0) than vice versa [20,30,31,34-36].

The designed heterogeneity in the groups of patients and observers is one of the sources of individual variations; uncertainty in interpretation of items another. Lack of understanding of a patient’s problem could also be an important factor of disagreement in patient-observer comparisons [20,32]. The measure of disorder, D, and the relative rank variance, RV, were calculated to evaluate the individual sources of disagreement. The proportion disordered pairs out of all possible different combination of pairs of assessments define, D [20,34]. For example, the pair *(no, moderate)* on the problem scale is disordered the pair *(slight, slight)* but both pairs agree in ordering with the pair (*moderate, large*). The RV is a rank-based variance measure of this variability. Possible values range between 0 and 1, but with five response categories, the maximum possible RV is 0.61 which is the RV when the pairs of data are uniformly distributed to all cells of the contingency table [20].

The evaluation of the patient-observer agreement refers to the paired assessments of the problems self-rated by the patients on the validated FB(2.0) and by the observers on the ICF-classification form. Presence of systematic disagreement, bias, between them was measured by the RP. A positive RP value indicates that patients more frequently assess higher levels of problems on the FB(2.0) than do the observers on the ICF-classification form than vice versa. Individual variability was expressed by the D and the RV. The measures of RP, RV and the corresponding 95% confidence intervals (CI) were calculated by a free software program [45,46].

## Results

The evaluation of construct validity was based on assessments on the FB(1.0) and the BIS made by 58 females and 31 males aged between 18 and 75 years; Md 48(Q_1_ 42; Q_3_ 58) years. The Spearman rank-order correlation coefficient of the relationship between the assessments on items of function and activity of the FB(1.0) and on similar items on the BIS ranged from 0.40 to 0.84, Table 2. The significant non-zero RP-values confirmed the systematic disagreement between the two operational definitions of the item scales of perceived problem and limitations in variables of function.

**Table 2:** Measures of agreement, disagreement and association in assessments on the items of function/activity in the Functional Barometer, FB(1.0) and on similar items on the Balanced Inventory for Spinal Disorders (BIS)

**The FB(1.0) items**	**The BIS items**	**n**	**RP**	**RV**
**Function/activity**	**Physical limitations**	**PA (%)**	**(95% CI)**	**(95% CI)**
		***r***_***s***_		**D**
Walking	Outdoor activities (shopping etc)	89	0.16	0.17
		36%	(0.04 to 0.28)	(0.08 to 0.26)
		r_s_ 0.45		0.14
Walking	Walking ability	87	-0.36	0.04
		32%	(-0.45 to -0.26)	(0.008 to 0.06)
		r_s_ 0.73		0.06
Walking up and down stairs	Walking ability	87	-0.37	0.09
		32%	(-0.47 to -0.26)	(0.03 to 0.14)
		r_s_ 0.61		0.10
Making the bed	Indoor activities (cleaning, cooking)	85	0.40	0.11
		28%	(0.30 to 0.51)	(0.04 to 0.17)
		r_s,_0.55		0.11
Going by car, bus, train	Outdoor activities (shopping etc)	87	0.23	0.22
		39%	(0.10 to 0.35)	(0.09 to 0.34)
		r_s_ 0.40		0.16
Going by car, bus, train	Leisure activities e.g. traveling, sports, societies	87	0.44	0.18
		34%	(0.33 to 0.54)	(0.07 to 0.29)
		r_s_ 0.47		0.14
Lifting, carrying a shopping bag	Outdoor activities (shopping, etc)	89	-0.06	0.13
		45%	(-0.17 to 0.05)	(0.02 to 0.25)
		r_s_ 0.53		0.10
Exercise/sports	Leisure activities e.g. traveling, sports, societies	89	0.05	0.09
		43%	(-0.05 to 0.16)	(0.03 to 0.16)
		r_s_ 0.56		0.10
Easy work	Indoor activities (cleaning, cooking)	86	0.41	0.18
		30%	(0.29 to 0.52)	(0.08 to 0.29)
		r_s_ 0.43		0.15
Sleeping in nights	I have sleeping disturbances	86	0.15	0.008
		53%	(0.077 to 0.22)	(0 to 0.02)
		r_s_ 0.84		0.02
Ordinary housework	Indoor activities (cleaning, cooking	87	0.37	0.06
		40%	(0.27 to 0.46)	(0.02 to 0.10)
		r_s_ 0.62		0.08

The measures of percentage agreement (PA), of the systematic disagreement in position (RP), of individual variability (RV), of disorder (D), and corresponding 95% confidence intervals (95% CI), and the Spearman rank-order correlation coefficient (r_s_).

The frequency distribution of the pairs of data from the assessments on the FB(1.0) item of perceived pain-related problem with *ordinary housework* and on the BIS item of pain-related limitations in *indoor activities* is shown in Figure 1. The significant, positive RP-value (0.37) indicates that the levels of problem with ordinary housework were somewhat smaller than the levels of limitation in indoor activities for the group of patients. The small RV-value (0.06) indicates slight individual variations which mean homogeneity in the group of assessments.

Corresponding set of measures hold for the variables of walking and walking up- and downstairs. The relationships between the perceived limitations on the BIS and the perceived problems with these variables on the FB(1.0) were confirmed by the r_s_, 0.73 and 0.61, respectively. The significant negative RP-values indicates that the these walking activities in the group of patients would be assessed to higher rather than lower levels of problems on the FB(1.0) when compared with the levels of limitations in walking ability on the BIS. The small RV- and D-values indicate slight additional individual variations.

The comparisons between the assessments of pain-related problems with quality-of-life variables on the FB(1.0) and corresponding assessments on the BIS show that the group of patients perceived lower levels of problem than of limitation effects according to the significant positive RP-values, Table 3. All but four item comparisons have a low D-value indicating homogeneity in assessments and fitness in item.

**Table 3:** Measures of agreement, disagreement and association in assessments on the items of quality-of-life in the Functional Barometer, FB(1.0) and on similar items on the Balanced Inventory for Spinal Disorders (BIS)

**The FB(1.0) items**	**The BIS items**	**n**	**RP**	**RV**
**Quality-of-Life**	**Quality-of-Life**	**PA (%)**	**(95% CI)**	**(95% CI)**
		**r**_**s**_		**D**
Work situation	To what extent do your pain complaints limit your way of living?	46	0.30	0.11
		43%	(0.15 to 0.44)	(0 to 0.23)
		r_s_ 0.53		0.10
Leisure time	Limitations in leisure activities	89	0.52	0.15
		21%	(0.42 to 0.62)	(0.07 to 0.23)
		r_s_ 0.42		0.14
Family situation	Limitations in social activities with your family. friends	89	0.59	0.20
		18%	(0.47 to 0.70)	(0.07 to 0.32)
		r_s_ 0.37		0.14
Relationship	Limitations in social activities with your family. friends	89	0.59	0.26
		17%	(0.47 to 0.72)	(0.10 to 0.41)
		r_s_ 0.26		0.17
Contacts with friends	Limitations in social activities with your family. friends	89	0.50	0.02
		25%	(0.40 to 0.59)	(0 to 0.05)
		r_s_ 0.56		0.04
Health	To what extent do your pain complaints limit your way of living?	88	0.27	0.11
		49%	(0.17 to 0.38)	(0.03 to 0.20)
		r_s_ 0.51		0.10
Insomnia	I have sleeping disturbances	87	0.17	0.001
		64%	(0.10 to 0.23)	(0 to 0.003)
		r_s_ = 0.84		0.004
Low. downheated	I feel down	89	0.14	0.07
		56%	(0.06 to 0.22)	(0.01 to 0.12)
		r_s_ 0.75		0.07
Stress tolerant	I feel impatient	87	0.11	0.20
		41%	(-0.01 to 0.23)	(0.06 to 0.34)
		r_s_ 0.50		0.14
Concentration difficulties	I have difficulty concentrating	88	0.06	0.03
		53%	(-0.02 to 0.13)	(0 to 0.06)
		r_s_ 0.80		0.05

The measures of percentage agreement (PA), of the systematic disagreement in position (RP), of individual variability (RV), of disorder (D), and corresponding 95% confidence intervals (95% CI), and the Spearman rank-order correlation coefficient (r_s_).

The perceived problems with pain were measured by four items with different response profiles. The median and quartiles of the level problem with *pain just now* were major (moderate; total) problem. Correspondingly major (moderate; major) problem with *pain during the last week*, total (moderate; total) problem with *worst pain*, and moderate (slight; moderate) problem with *mildest pain* respectively.

### The validated functional barometer

The result of the validation study of the FB(1.0) mainly concerned the verbal description of the items and the order consistency in the inter-item comparisons to similar variables of the BIS, and resulted in a major reformulation of the items to clarify that they referred to the patient’s own problem to perform an activity without help. The fitness of the formulation of variables to the ICF variables was also improved. Hence, the operational definitions of the variables of the revised Functional Barometer meet the requirements of good construct and content validity.

The validated FB(2.0) consists of 29 items which verbally describes the problem to be measured, for example: *Do you have problems making your bed because of your pain?*

The problem scale categories are: *no, slight, moderate, major,* and *total problems.* The variables of body function activity/participation are measured by 12 specified items and one open question asking for “*another activity that brings you problems because of your pain*”. Perceived problems with quality-of-life variables and with pain are measured by 12 and four items respectively. The item variables and the corresponding ICF codes are seen in Tables 4 and 5.

**Table 4:** Measures of agreement, systematic disagreement (bias) and individual variability in pain-related problems with function between patient’s assessments on the validated Functional Barometer, FB(2.0) and the observer’s ICF-classifications

**The FB(2.0) items of function and corresponding ICF codes**	**n**	**PA (%)**	**RP (95% CI)**	**RV (95% CI)**	**D**
Do you have problem because of your pain with….					
Dressing	41	32%	-0.19	0.32	0.20
(ICF d 540)			(-0.39 to 0.02)	(0.09 to 0.55)	
Joint mobility	41	29%	-0.16	0.36	0.21
(ICF b 710)			(-0.38 to 0.05)	(0.07 to 0.64)	
Muscle strength	39	18%	-0.29	0.35	0.20
(ICF b 730)			(-0.51 to -0.07)	(0.07 to 0.63)	
Endurance	39	23%	-0.17	0.38	0.23
(ICF b 740)			(-0.41 to 0.06)	(0.12 to 0.64)	
Walking	40	33%	-0.31	0.20	0.14
(ICF d 450)			(-0.49 to -0.13)	(0.002 to 0.40)	
Walking in stairs	41	51%	-0.11	0.29	0.19
(ICF d 4551)			(-0.29 to 0.07)	(0.07 to 0.52)	
Keeping posture	41	22%	-0.60	0.15	0.13
(ICF d 4153)			(-0.75 to -0.45)	(0.02 to 0.27)	
Making the bed	40	30%	0.01	0.27	0.19
(ICF d 649)			(-0.19 to 0.22)	(0.08 to 0.47)	
Ordinary housework	41	29%	-0.27	0.09	0.11
(ICF d 640)			(-0.43 to -0.11)	(0.02 to 0.17)	
Lifting/carrying things	39	28%	-0.30	0.40	0.24
(ICF d 430)			(-0.53 to -0.07)	(0.13 to 0.68)	
Driving a car	39	33%	-0.06	0.33	0.22
(ICF d 4751)			(-0.28 to 0.15)	(0.09 to 0.57)	
Using transportation	41	29%	-0.46	0.31	0.21
(ICF d 470)			(-0.65 to -0.27)	(0.11 to 0.52)	

The measures of percentage agreement (PA), the systematic disagreement in position (RP), individual variability (RV), disorder (D), and corresponding 95% confidence intervals (95% CI).

**Table 5:** Measures of agreement, systematic disagreement (bias) and individual variability in pain-related problems with quality-of-life and pain between patient’s assessments on the validated Functional Barometer, FB(2.0) and the observer’s ICF-classifications

**The FB(2.0) items of quality-of-life and corresponding ICF codes**	**n**	**PA (%)**	**Systematic disagreement (bias)**	**Individual variation**	**D**
			**RP (95% CI)**	**RV (95%CI)**	
Do you have problem because of your pain with….					
Sleeping	41	27%	-0.47	0.08	0.09
(ICF b 134)			(-0.61 to -0.33)	(0 to 0.16)	
Energy	38	34%	-0.38	0.25	0.17
(ICF b 130)			(-0.57 to -0.19)	(0.03 to 0.48)	
Concentration	41	20%	-0.44	0.38	0.24
(ICF b 160)			(-0.64 to -0.24)	(0.14 to 0.62)	
Stress. psychological demands	41	32%	-0.30	0.30	0.20
(ICF d 240)			(-0.51 to -0.09)	(0.08 to 0.52)	
Emotional functions	39	36%	-0.32	0.27	0.20
(ICF b 152)			(-0.51 to -0.13)	(0.08 to 0.47)	
Gastro-intestinal functions	41	37%	-0.25	0.23	0.18
(ICF b 535)			(-0.44 to -0.06)	(0.06 to 0.41)	
Leisure time	40	28%	-0.65	0.15	0.13
(ICF d 920)			(-0.80 to -0.51)	(0.02 to 0.27)	
Family relation	40	25%	-0.47	0.27	0.18
(ICF d 760)			(-0.66 to -0.28)	(0.06 to 0.47)	
Partner relationship	29	38%	-0.38	0.26	0.19
(ICF d 770)			(-0.60 to -0.16)	(0.04 to 0.47)	
Contact with friends	40	35%	-0.54	0.12	0.11
(ICF d 750)			(-0.70 to -0.39)	(0 to 0.25)	
Self-support	40	25%	-0.13	0.20	0.16
(ICF d 750)			(-0.32 to 0.06)	(0.03 to 0.37)	
Managing pay-work	29	38%	-0.25	0.33	0.22
(ICF d 850)			(-0.48 to -0.02)	(0.06 to 0.59)	
**Perceived pain**					
Pain just now	41	44%	-0.40	0.14	0.12
(ICF b 280)			(-0.55 to -0.24)	(0 to 0.29)	
Pain the last week	41	37%	-0.38	0.05	0.06
(ICF b 280)			(-0.51 to -0.24)	(0 to 0.09)	
The mildest pain	41	34%	-0.28	0.14	0.12
(ICF b 280)			(-0.47 to -0.10)	(0.01 to 0.27)	
The worst pain	41	46%	0.00	0.23	0.19
(ICF b 280)			(-0.22 to 0.22)	(0.04 to 0.43)	

The measures of percentage agreement (PA), the systematic disagreement in position (RP), individual variability (RV), disorder (D), and corresponding 95% confidence intervals (95% CI).

The patient-observer comparison of the FB(2.0) and the ICF assessments involved 25 females and 16 males aged between 24 and 76 years; median 46, (Q_1_ 36; Q_3_ 58) years. The origin of the pain varied but the majority of the patients suffered from pain from the musculoskeletal system, such as fibromyalgia, low back pain and localized pain.

**Figure 2:**
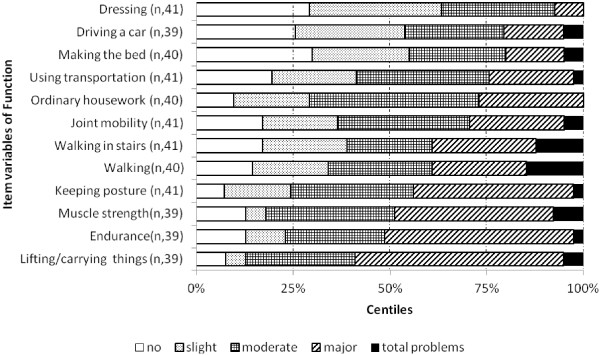
**The assessment profile of perceived problems regarding items of function on the validated Functional Barometer, FB(2.0).** The centiles of the median and quartiles are shown.

**Figure 3:**
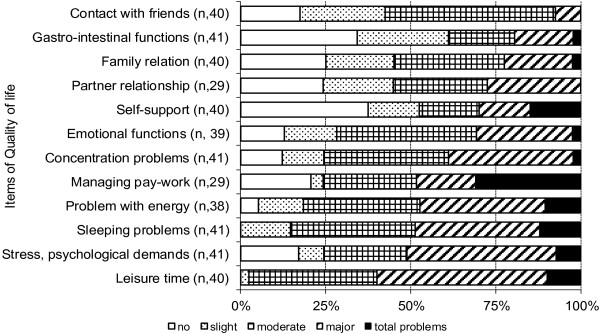
**The assessment profile of perceived problems regarding the items of quality-of-life on the validated Functional Barometer, FB(2.0).** The centiles of the median and quartiles are shown.

The assessment profile of perceived problems with the specified variables of body function/activity showed that about 50% of the patients rated major or total problems with *lifting and carrying things*, *muscle strength* and *endurance*, Figure 2. Correspondingly, *leisure time*, *stress and psychological demands* were the quality-of-life variables that caused at least major problems in about half of the patients, Figure 3.

**Figure 4:**
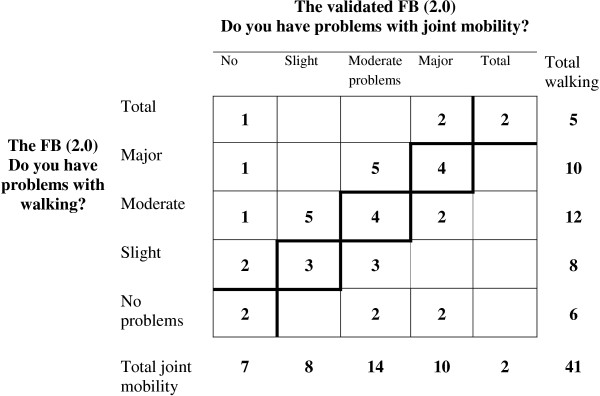
**The frequency distribution of the pairs of assessments on the Functional Barometer, FB(2.0) of perceived problems with joint mobility and of walking.** The agreement diagonal is marked.

The frequency distributions of the patients’ assessed levels of problem with the FB(2.0) items *joint mobility* and of *walking* were similar with the same median and third quartiles, moderate and major problems, respectively, but the paired distribution of the assessments shows the discriminating ability of these items, Figure 4. Ten patients have used the category major problems in both assessments but only four of them represent the same patients having major problems with both variables. The PA is 37% and the main reason for disagreement is the individual variability, D = 0.19.

### Self-report assessments on the validated FB versus ICF-classifications by observers

The observers’ ICF-classifications of the patients’ problems disagreed with the patients’ assessments on the FB(2.0), the median percentage agreement was 32%, (Range 18% to 51%), see Table 4 and Table 5. The main reason for the disagreement was that the observers systematically underestimated the patients’ levels of problems, as evident from the statistically significant negative RP-values. The different professionals contributed to the large individual variability, the median RV being 0.26 (Range 0.05 to 0.40), and D 0.19 Range 0.06 to 0.24).

**Figure 5:**
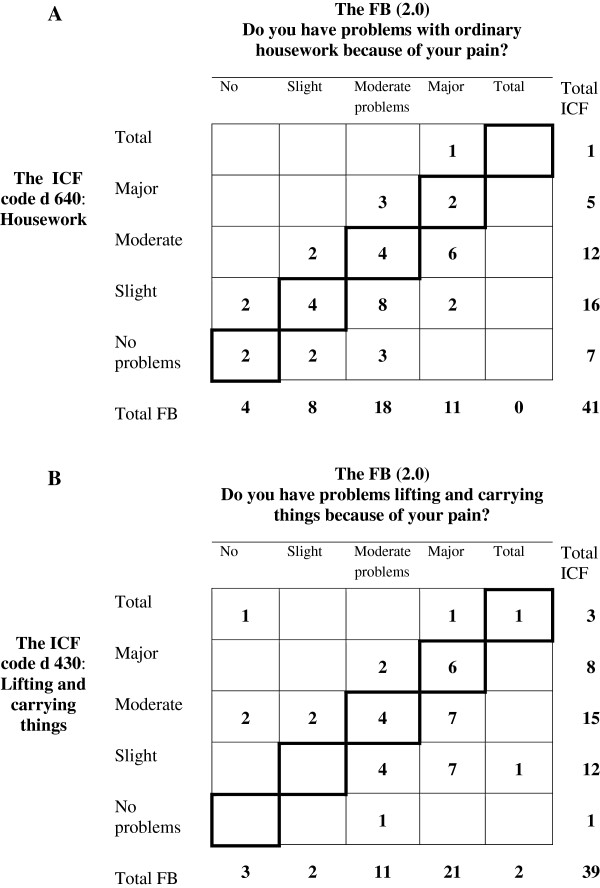
**The frequency distributions of patients’ assessments on the Functional Barometer, FB(2.0) versus observers’ ICF-classifications regarding the patients’ pain-related problems with ordinary housework (A), and with lifting and carrying things (B).** The agreement diagonals are marked.

The paired assessments of problems with ordinary housework (ICF d640) and with lifting and carrying things (ICF d430) had similar levels of agreement, PA, 29% and 28%, respectively, but the reasons for disagreements differ. In the assessments of patients’ problem with ordinary housework the observers systematically used lower categories than did the patients, RP -0.27, Figure 5A. One observer contributed strongly by assessing nine patient as having *no* or *slight problems* while these patients’ assessed *no, slight, moderate* and *major problems.*

In the assessments of lifting and carrying the heterogeneity among observers, RV 0.40, was the main reason for disagreement. The disagreeing pairs (*no, total problems*) and (*total, slight problems*) assessed by two different observers contributed with 34% to the RV-value, Figure 5B.

The patients’ problems with the quality-of-life and pain variables were systematically classified to lower categories by the observers. Except for the variables self-support (ICF d 750) and the worst pain the RP-values range between –0.25 and –0.65. The 95% confidence intervals of these significant negative RP-values are strong evidences that the observers’ ICF-classifications of problems in a relevant population of patients more likely will be lower than the self-rated levels of problems on the FB(2.0) than the opposite.

## Discussion

The aim of this study was to evaluate the Functional Barometer as being an ICF-based self-report valid questionnaire for assessments of perceived pain-related problems with functions, activity and quality-of-life by patients suffering from long-term pain. The content and construct validity studied refer to the operational definition of the item variables and will be generally valid and independent of the application. Our study resulted in a revised FB(2.0) with valid properties.

As mentioned, quality control of questionnaires and of data from scale assessments is an ongoing process and most of the three components: validity, reliability and responsiveness refer to specific contexts, types of studies and/or types of applications and target populations [20]. The Functional Barometer is so far only validated regarding the content and construct validity of the Swedish version, http://www.funktionsbarometern.se. When translated to other languages the translation validity must be considered. Concepts of reliability are study specific. The test-retest reliability, also called the intra-rater reliability, must be evaluated when introducing a questionnaire to an empirical study in order to establish the stability of assessments [20,47,48]. In clinical studies of treatment effects or other types of follow-up studies the outcome measures must be reliable, which means that the assessments must be sensitive to changes. Then the responsiveness to changes must be evaluated [20,49-51].

We have used a statistical method that takes account of the non-metric properties of ordered categorical data. Such data have become very common in studies because of the increased interest in qualitative variables and probably also because of the WHO recommendation of quality-of-life assessments in research and clinical practice. The statistical method used [20] is the only method designed for a comprehensive evaluation of disagreement in paired ordinal data by providing separate measures of systematic disagreement and the additional individual variability.

The designed heterogeneity in the groups of patients and observers was one expected source of individual variability in this study, possible uncertainty interpretation of items another. The paired assessments on the FB(1.0) and the BIS showed that the main reason for the observed disagreement was the systematic disagreement because of different operational definitions of the items and not the heterogeneity in patients. The observed disagreements in the assessments on the FB(1.0) vs the BIS, referring to exercise vs leisure activities (Table 2), and to the work situation vs the way of living (Table 3) showed the same agreement of 43%, but the main reason for disagreement in the first comparison was the slight individual variability and in the second an additional significant systematic disagreement indicating the differences between the operational definitions of the items.

Previous inter-observer clinical studies have shown systematic disagreement in assessments out of different levels of skills or perspectives, findings that motivated the design of this study. In a study on children with juvenile chronic arthritis the parent systematically overestimated the child’s pain compared with the child’s assessment on a four-point verbal descriptive scale [32]. In another study, children who were undergoing a long-term hormone treatment evaluated a new device, and so did the parents and the nurse. Both the treatment nurse and the parents systematically underestimated the children’s positive perceived assessments of the new device. No such systematic disagreement was found between the nurse and parents [52]. In neuroradiology less experienced radiologists underestimated the severity of brain damage when compared with the assessments made by the expert which could be fatal for the patients’ life [31].

In accordance with the WHO Guideline and the suggested use of the ICF framework and core sets for obtaining an integrated deeper understanding of the patient’s problems and need, the self-report FB and the corresponding ICF-classification form for professional’s assessments were developed [18,22,27,29]. Since the professional’s understanding of a patient’s pain-related functioning state is important for the rehabilitation process, for decisions about the patient’s needs, for support in daily life, a comparison of the two different perspectives of the patient’s perceived problems (patient vs observer) was of great interest. The statistical evaluation revealed that the assessments made by the professionals significantly underestimated the patients’ perceived problems. Also a large variability between the different observers was found. These findings might lead to a better understanding of the value of integrating the patient’s perception of pain-related problems with the professional’s assessments and could have an impact on the patient’s rehabilitation in the future.

## Conclusions

Having the study specific concepts of quality control of questionnaires in mind, the Functional Barometer is a valid ICF-based self-report questionnaire that could be used for different purposes regarding assessments of current functioning, activities and follow-up assessments after treatment or rehabilitation. Our results clearly demonstrated the need of the self-report FB(2.0) for assessment of the level of pain-related problems, since the patient’s perspective will provide important complementary information benefiting treatment and rehabilitation.

## Competing interests

The authors declare that they have no competing of interests.

## Authors’ contributions

JRN developed the FB and drafted parts of the manuscript. ES carried out the study designs, the statistical evaluations, participated in the operational definitions of the final version of FB-items, and drafted the manuscript. Both authors read and approved the final manuscript.

## Pre-publication history

The pre-publication history for this paper can be accessed here:

http://www.biomedcentral.com/1472-6963/14/187/prepub
